# A Transformable Sheet Type Robot That Can Be Thrown from the Air

**DOI:** 10.3390/biomimetics7030114

**Published:** 2022-08-16

**Authors:** Naoki Iida, Mitsuharu Matsumoto

**Affiliations:** Graduate School of Informatics and Engineering, University of Electro-Communications, 1-5-1, Chofugaoka, Chofu-shi, Tokyo 182-8585, Japan

**Keywords:** sheet type robot, deformable robot, robot control, system implementation

## Abstract

This paper reports on a transformable sheet type robot that can be thrown from the air. Since sheet type robots can change their own shape and perform tasks according to the situation, they are expected to play an active role in situations with many restrictions, such as disaster-stricken areas. However, since most sheet type robots jump or crawl on the ground, the only way to deliver them to the site of a disaster is to transport them by vehicle or transporter. This research aims to develop a device that can be dispersed from the sky and perform activities on the ground after landing.

## 1. Introduction

So far, various studies on deforming robots have been reported. Some studies have developed deformable robots with several modes that change mobility in response to changes in the environment. One example is a hybrid robot with a wheel mode for land movement and a helicopter mode for aerial movement [1]. Another example is a report on a robot capable of moving in the air and on the ground [2]. On the other hand, research on modular self-reconfigurable robots (MSR) is also actively being conducted. According to the survey on MSRs [3], early research on MSRs began in the 1980s [4]. Since then, many studies on MSRs have been reported. Yim et al. proposed a modular type robot named Pollybot [5]. Gilpin et al. proposed a lattice type robot named Crystalline system [6]. Murata et al. proposed a modular robot named M-TRAN [7,8,9].

Many MSRs transform by mechanically changing the connections between the modules. Hence, the modules tend to become relatively large and complex. Therefore, it is necessary to consider the positioning problem when connecting modules to each other. The size of the module itself also becomes large and complicated.

Another approach to transforming robots is research on sheet type robots that can transform two-dimensional planes into various three-dimensional shapes. Sheet type transformable robots have several advantages over other transformable and modular robots. Various shapes can be formed from a flat sheet and folded to make the occupied volume very small.

Several related studies on sheet robots have been reported so far. For example, Hawkes et al. reported a sheet type material named programmable matter [10,11]. In this study, shape memory alloys and magnets were used for the transformation from a two-dimensional plane to a three-dimensional shape. Another related study is the field of self-organization. An example is a three-dimensional closed circuit from a sheet using magnetic force [12].

In recent years, various studies on disaster response robots have been conducted. These robots are used to rescue victims of earthquakes and floods [13,14,15,16,17].

Quick exploration activities are one of the important activities in the event of a disaster. There are two types of robotic disaster exploration: an aerial exploration approach and a land-based exploration approach.

Unmanned aerial vehicles (UAVs) are a useful method for exploration from the sky.

According to [18], unmanned aerial vehicle can be categorized into three types.

The first approach uses one or more aerodynamic surfaces as fundamental lift sources accompanied by propulsion systems [19,20,21].

In the second approach, vehicles have upward-pointing single or multiple fixed-pitch propellers with no other aerodynamic lift [22,23].

In the third approach, the robots have biomimetic soft flapping-wings [24,25].

UAVs are useful for exploring from the sky.

However, many UAVs are battery-powered, so they can only fly for a short time. UAVs are vulnerable to strong winds and bad weather as they fly in the sky and continue exploration. On the other hand, in order to conduct exploration on the ground, it is necessary to carry the robot to the site.

To solve the above problems, the following two-step approach is assumed as a method for quickly deploying a large number of disaster support robots to the disaster area in this research. First, a large number of robots are thrown into a disaster area using an unmanned aerial vehicle such as a helicopter or a drone. They then move on the ground by their own driving force. To achieve this goal, we report in this study on a framework that automatically switches from flight mode to walking mode. When the robots are actually used, they are mainly controlled in walking mode. Based on the above prospects, we have adopted a simple and safe passive operation for the flight mode. On the other hand, it is possible to control the walking mode after falling.

To achieve this goal, we pay attention to sheet type robots.

Sheet type robots are also attracting attention as disaster rescue robots. The ability of sheet-type robots to transform from a two-dimensional structure into a variety of three-dimensional structures offers the possibility of both adaptability to various environments in disaster-stricken areas and the ability to reduce size and cost [26,27].

Many disaster robots are focused on use at the disaster site, so they need to be transported to the disaster site. On the other hand, as sheet type robots are lightweight and softer than other robots, if they can be safely thrown from the sky, they can be quickly placed at the disaster site. In this study, we pay attention to the softness and lightness of the origami robot and investigate a sheet type robot that can be thrown from the sky and then move on the ground.

The rest of the paper is organized as follows: The next section describes the design of a robot that can be thrown from the sky. This study examines a robot that imitates the winged fruit of a plant to reduce the falling speed of the robot.

We investigate three plants as mimicking plants and formulate a physical model for the most promising dipterocarp. In Section 3, we describe a sheet type transformable robot that can both fall by imitating dipterocarp seeds and move on the ground by imitating inchworms. We also conduct a drop experiment and a movement experiment using the developed robot. The performance is quantitatively evaluated through the experiment. Section 4 describes the discussion from the experimental results. Conclusion and future works follow in Section 5.

## 2. Robot Design That Mimics the Winged Fruit of Plants

### 2.1. Wing Effect of Winged Fruit of Plants

In this study, we consider imitation of plants as a method of throwing robots from the air. Some plants disperse their fruits by the power of wind. Wind-scattering winged fruits have a special shape, and when they fall from the tree, they speed down in the air and move with the wind. Many of these motions can be reproduced with paper or plastic sheets, and are highly compatible with sheet type robots.

Unlike the parachute method, the fall by a plant does not use a string. Therefore, even if there are many robots, it is expected that the robots can be dropped. In addition, there is no need to manipulate or operate the device in the air, and the dropping speed can be reduced by simply dropping the device.

**Figure 1:**
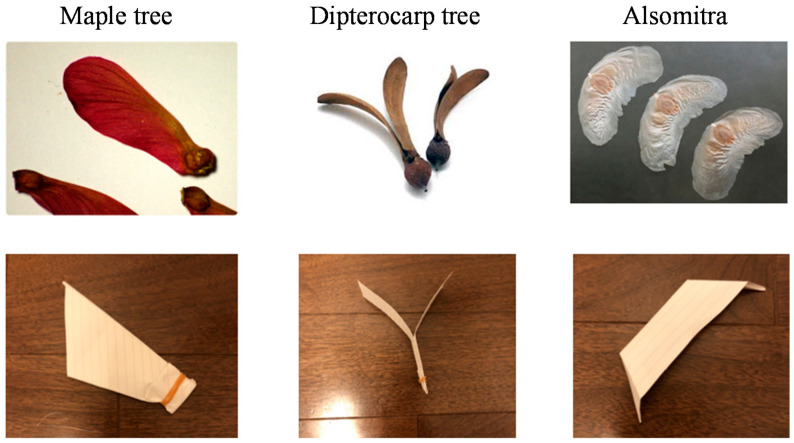
Model of wing fruit.

**Figure 2:**
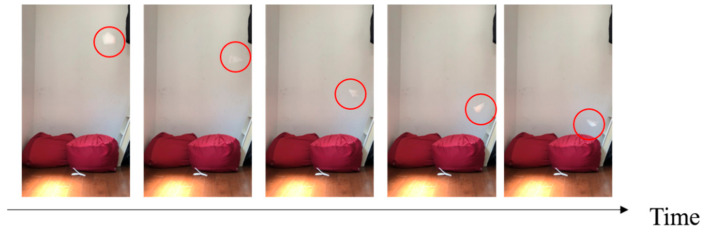
Falling test of the model of the maple tree.

**Figure 3:**
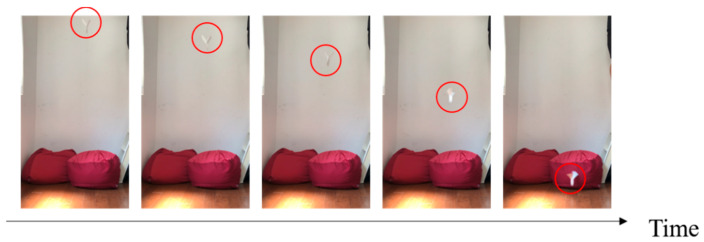
Falling test of the model of the dipterocarp.

**Figure 4:**
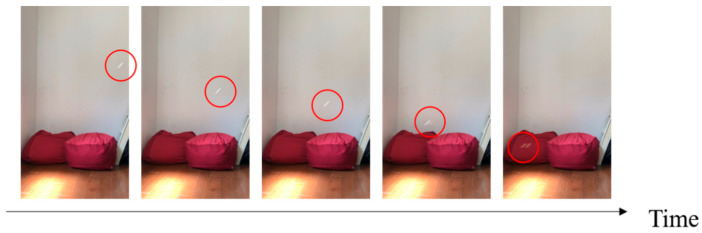
Falling test of the model of the Alsomitra.

We investigated three candidates for mimicking plants: maple trees, dipterocarp trees, and Alsomitra. Figure 1 shows photographs of the original plants and their models that mimic them. Figure 2, Figure 3 and Figure 4 show the falling test of the models of maple trees, dipterocarp trees, and Alsomitra, respectively. The model in the figures is circled in red for clarity. Although the maple model has a simple structure, it could fall without rotating depending on how it is dropped. Since the Alsomitra model glides like a paper airplane, it is difficult to land it on the target point from the sky. On the other hand, the dipterocarp model rotates easily and stably. From these results, we chose the dipterocarp model as the flight model for our device.

### 2.2. Falling Model of the Dipterocarp Model

In order to realize the stable rotation and fall of the dipterocarp model, we examine the physical model and consider what determines the rotation speed and fall speed.

#### 2.2.1. Energy Conservation Law

Let us consider the situation where the robot is dropped from height *H* to *h*. The law of conservation of energy when the model falls from height *H* to height *h* while spinning is expressed by the following equation.

(1) $$MgH=\frac{1}{2}MV^{2}+\frac{1}{2}I\omega^{2}+Mgh,$$

where the mass of the model is *M*. The falling velocity is *V*. The moment of inertia of the wings is *I*. The angular velocity of the spinning wings is ω. From Equation (1), the fall velocity *V* can be expressed as follows:

(2) $$V=\sqrt{\;2g\left(H-h\right)-\frac{I\omega^{2}}{M}}$$

From Equation (2), the fall velocity *V* becomes larger as the moment of inertia *I* and the angular velocity *ω* become smaller, and it becomes smaller as the mass *M* becomes smaller. The moment of inertia *I* can be expressed by the following equation using a mass $m_{i}$ at distance $r_{i}$ from the axis of rotation.

(3) $$I=\sum_{i}m_{i}r_{i}$$

From this equation, the larger the weight $m_{i}$ of the feather in the part far from the axis of rotation, the larger the moment of inertia.

#### 2.2.2. Mechanism of Rotation

**Figure 5:**
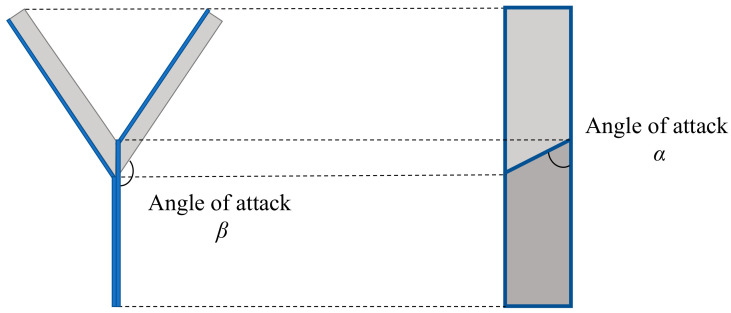
Elevation angle and angle of attack.

Figure 5 shows the elevation angle and angle of attack of the dipterocarp model. As shown in Figure 5, a wing of mass m has two angles of attack *α* and *β*. When it starts to fall, the wing is pushed up by the air and obtains drag force *d* and lift force $l_{\alpha }$ and $l_{\beta }$ (0 < *α* < *π*/2 < *β* < *π*).

**Figure 6:**
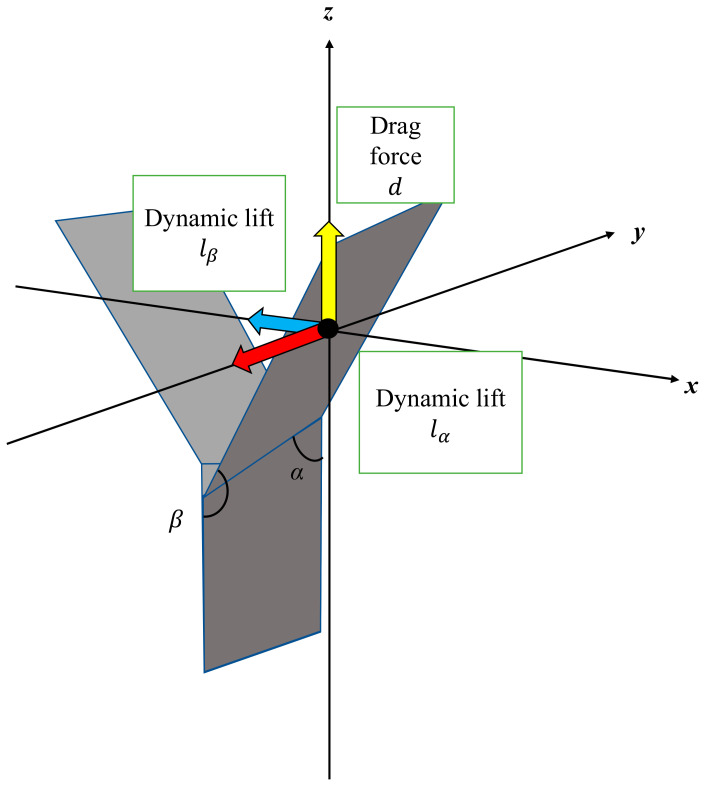
Forces acting on the model.

**Figure 7:**
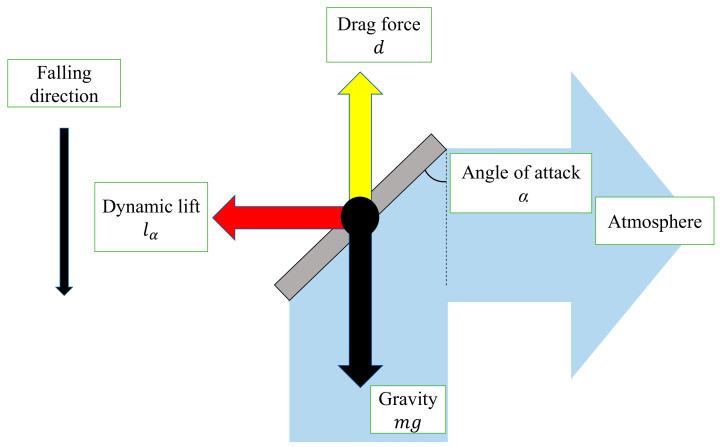
Cross-section of wing, lift force *l*_α_ with angle of attack α.

Figure 6 shows the forces acting on the model. Figure 7 shows the relationship between the cross-section of the wing and the lift force due to the angle of attack *α*.

**Figure 8:**
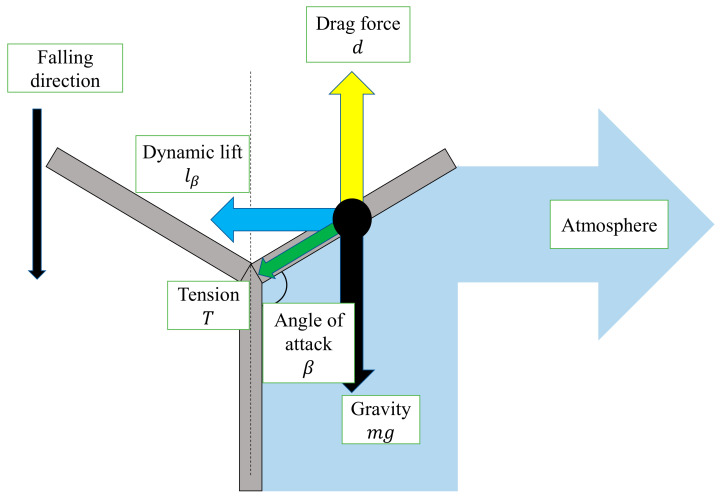
Lift force *l_β_* due to angle of attack *β*.

Figure 8 shows the relationship between the wing inclination and the lift force due to the angle of attack *β*. When the wing pushes the air away from it, the lift force $l_{\alpha }$ is obtained in the tangential direction of the circular motion and the model rotates. The lift force $l_{\beta }$ acts as one of the centripetal forces.

#### 2.2.3. Drag Force and Lift Force

The drag force *d* is expressed as follows, with the drag coefficient $C_{d}$, the wing area as the representative area *S*, the density of the atmosphere *ρ*. Here, the atmosphere is stationary, and the representative velocity is assumed to be equal to the falling velocity *V*.

(4) $$d=\;\frac{C_{d}S\rho V^{2}}{2}$$

**Figure 9:**
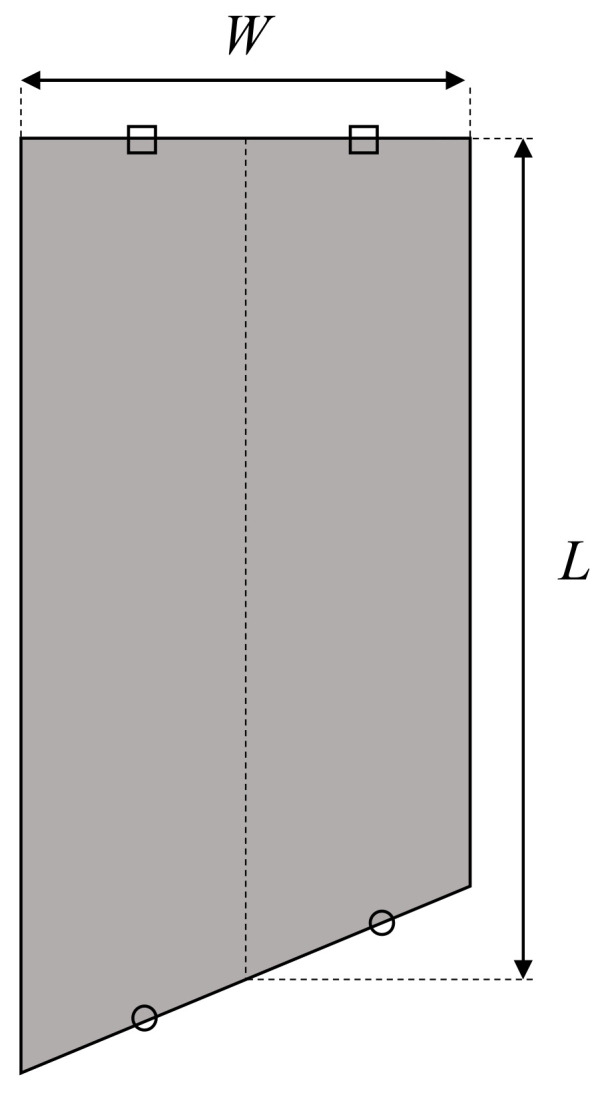
Representative area.

The lift forces *l_α_* and *l_β_* by *α* and *β* are respectively expressed as follows.

(5) $$l_{\alpha }=\;\frac{C_{l_{\alpha }}S\rho V^{2}}{2}$$

(6) $$l_{\beta }=\;\frac{C_{l_{\beta }}S\rho V^{2}}{2}$$

where $C_{l_{\alpha }}$ and $C_{l_{\beta }}$ are the coefficient of the lift forces *l_α_* and *l_β_*, respectively. As shown in Figure 9, if the length of the wing is *L* and the width is *W*, the representative area *S* is as follows:

(7) S=WL

Therefore, *d* can be expressed as follows.

(8) $$d=\;\frac{C_{d}WL\rho V^{2}}{2}$$

*l_α_* and *l_β_* are respectively expressed as follows:

(9) $$l_{\alpha }=\;\frac{C_{l_{\alpha }}WL\rho V^{2}}{2}$$

(10) $$l_{\beta }=\;\frac{C_{l_{\beta }}WL\rho V^{2}}{2}$$

The drag coefficient $C_{d}$ is a non-dimensionalization of the drag force *d* in terms of dynamic pressure and representative area, and becomes larger as the angle of attack *α* increases and smaller as the angle of attack *β* increases. (0 *< α < π*/2 *< β < π*). The dynamic pressure is the kinetic energy of the fluid per unit volume converted into pressure.

On the other hand, the lift coefficient $C_{l_{\alpha }}$ is a dimensionless quantity. It increases as α increases when the angle of attack α is small, but it decreases after a certain angle (0 *< α < π*/2). It also becomes smaller as the angle of attack *β* becomes larger (*π*/2 *< β < π*).

The lift coefficient $C_{l_{\beta }}$ is a dimensionless quantity. It increases as *β* increases when the angle of attack *β* is small, but it decreases after a certain angle *(π*/2 *< β < π*). It also becomes smaller as the angle of attack *α* becomes larger (*π*/2 *< α < π*).

#### 2.2.4. Equation of Motion

**Figure 10:**
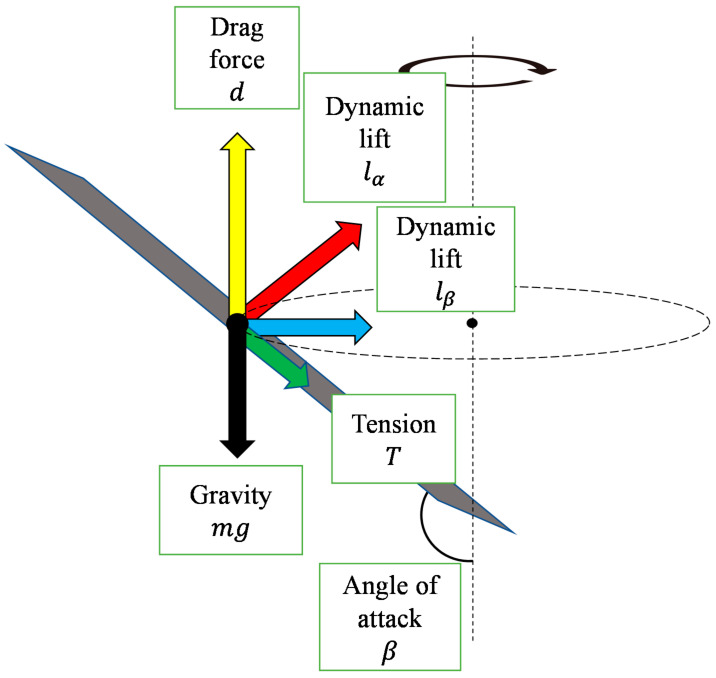
Rotational motion of a single wing.

Figure 10 shows a schematic diagram of the rotational motion of a single wing. Since *β* is stable during the rotational fall, the following equation of balance can be obtained.

(11) $$d=mg+T\cos\left(\pi -\beta \right)$$

From the equation of motion for rotation, the following equation can be obtained.

(12) $$m\frac{L}{2}\sin\left(\pi -\beta \right)\frac{d\omega }{dt}=l_{\alpha }$$

(13) $$m\frac{L}{2}\sin\left(\pi -\beta \right)\omega^{2}=l_{\beta }+T\sin\left(\pi -\beta \right)$$

Equation (12) is the equation of motion in the tangential direction. Equation (13) is the equation of motion in the central direction. Integrating both sides of the Equation (12) with *t* and transforming the equation, we obtain the following equation.

(14) $$\omega =\frac{C_{l_{\alpha }}\;\;W\;\rho V^{2}t}{m\sin\beta }$$

By transforming Equation (13) using Equations (8), (10) and (11), *ω* in the case of constant velocity circular motion can also be expressed as follows

(15) $$\omega =\sqrt{\frac{2\left(C_{l_{\beta }}\cos\beta -C_{d}\;\sin\beta \right)W\rho V^{2}}{m\sin2\beta }+\frac{2g}{L\cos\beta }}$$

In the case of constant velocity circular motion, the values of Equations (14) and (15) are equal.

Although the angle of attack *β* is one of the variables that determine *ω*, it also affects *I*, $C_{d}$, $C_{l_{\alpha }}$, $C_{l_{\beta }}$, and so on. We formulate the dynamics of the robot by using several physical formulas. However, some parameters such as $C_{d}$, $C_{l_{\alpha }}$ and $C_{l_{\beta }}$ appearing in the equation are determined experimentally. From Equation (14), we decided to adjust the value of *α* to control the robot speed more slowly and conduct the experiments to find at what value of *α* the falling velocity *V* can be reduced.

## 3. Sheet Type Transformable Robot

### 3.1. Design of Sheet Type Transformable Robot

In this section, we describe our developed sheet type transformable robot.

For the sheet type transformable robot, we used PP sheets. They can easily be deformed and have the smallest density among plastics (0.9–0.91 g/cm^3^). These features make them suitable for throwing from the air. To drive the robot, we used an actuator made of a shape memory alloy called Biometal made by the Toki Corporation. Biometal is a linear actuator and contracts when an electric current is passed through it. When the current is stopped, it will stretch to its original length and can be moved repeatedly. Biometal has two types of actuators, Biometal Fiber (BMF) and Biometal Helix (BMX). BMX was used this time as BMX has a higher expansion ratio than BMF.

Although PP has relatively high heat resistance among general-purpose plastics, its melting point is between 100 and 140 degrees Celsius. Therefore, we reduced the contact surface between the PP plate and BMX by using round terminals to prevent melting from the heat generated when electric current is applied to the BMX. In order to adjust the friction surface during walking, we used steel wires to fix the angle of the legs. As a result of modeling, it was found that *α* is important as a parameter that determines the speed. Therefore, adequate *α* was determined through the falling experiments. Other parameters were determined experimentally in consideration of the ease of walking after falling from the sky.

### 3.2. Experiment of Adequate Elevation Angle

We conducted a drop experiment to find the adequate elevation angle *α* as it affects the optimum lift coefficient $C_{l}$ to reduce the fall velocity. The elevation angle *α* was changed by π/12 (rad) from 2π/12 (rad) to 5π/12 (rad).

To measure the falling speed and walking speed of the robot, we first recorded the falling and walking of the robot as a video. The falling and walking speeds were measured by performing image analysis on the recorded video. In the falling experiment, the rotation speed and falling speed of the robot were measured. Regarding the rotation speed, a black marker was attached to the wing to measure how many times it rotated within a certain period of time. Regarding the falling speed, we set the fall distance in advance and analyzed how long it took to fall the specified distance with a video.

**Figure 11:**
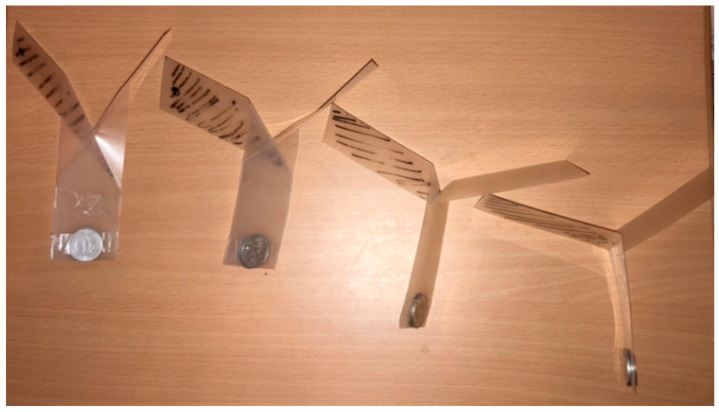
Model used in the experiment.

The actual configuration of the sheet type transformable robot is shown in Figure 11. The drop velocity and angular velocity were measured from a height of 2 m. The results are shown in Table 1. The results show that the angular velocity was maximum when the elevation angle *α* was 4π/12 (rad). However, the velocity was the lowest when *α* was 5/12 π (rad). This may be due to the effect of the change in moment *I* caused by the change in elevation angle *β* due to the drag force *d*, or because the drop velocity *V* is included in the equation to calculate the angular velocity ω, and the angular velocity becomes smaller when the drop velocity becomes smaller. From the results of the experiment, *α* = 5/12 π is considered to be the best for the purpose of reducing the fall velocity. However, when *α* = 5/12 π, the robot could not take a rotating posture when it was dropped upside down. For the purposes of this research, it is desirable to be able to take a rotating posture no matter what posture the object is dropped from. Hence, we decided to use *α* = 4/12 π this time.

### 3.3. Transformation Mechanism from Falling Form to Walking Form

**Figure 12:**
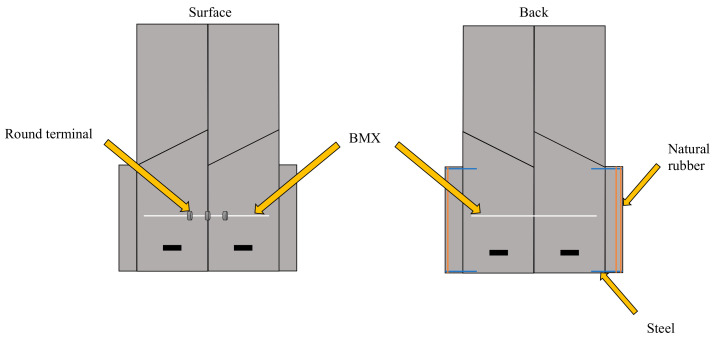
Components of sheet type robot.

In order to transform the robot from a falling form to a walking form quickly, we designed the robot to transform by separating a part of the robot by the impact of falling. Figure 12 shows a schematic diagram of the implementation.

**Figure 13:**
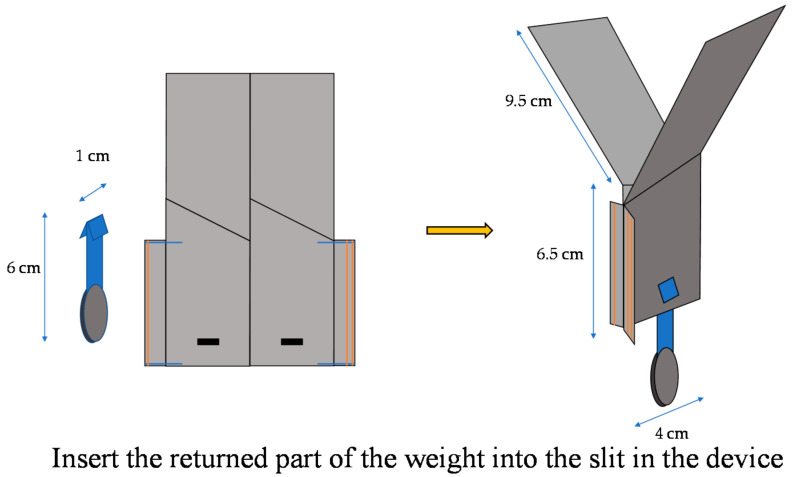
Falling form by weight return. Regarding the main body, its weight is 4.1 g. Fuselage length and width are 6.5 cm, and 5 cm, respectively. Wing length and width are 9.5 cm and 4 cm, respectively. Regarding a weight with a return, its weight is 2.3 g. Its length and width are 6 cm and 1 cm, respectively.

As shown in Figure 13, when the robot falls, it maintains a stable falling posture by hanging a weight with a return. The specification of the robot is also shown in Figure 13. Regarding the main body, its weight is 4.1 g. Fuselage length and width are 6.5 cm, and 5 cm, respectively. Wing length and width are 9.5 cm and 4 cm, respectively. Regarding a weight with a return, its weight is 2.3 g. Its length and width are 6 cm and 1 cm, respectively.

**Figure 14:**
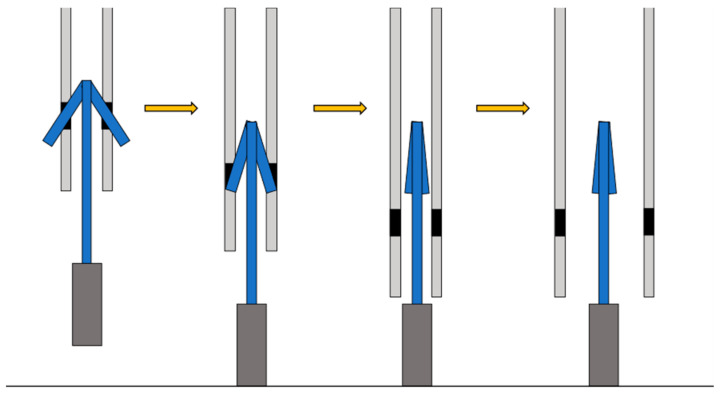
Deformation caused by the impact of falling.

As shown in Figure 14, when the robot falls to the ground, the impact of the fall pushes the weight back upward, separating the weight and opening the device, which is no longer restrained, into a walking form.

### 3.4. Walking Mechanism

**Figure 15:**
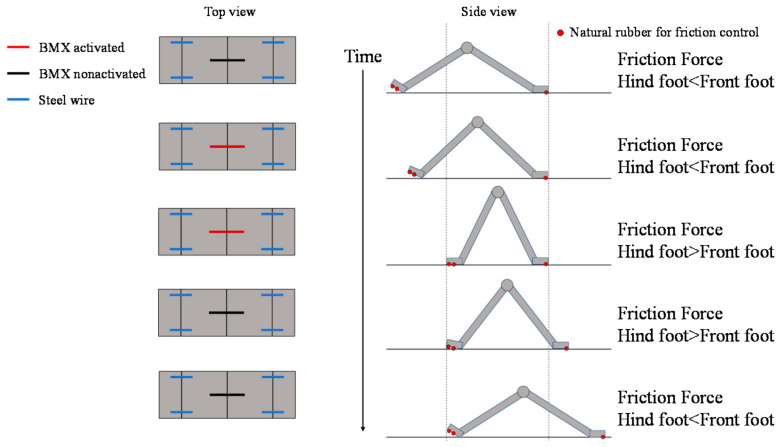
Walking mechanism of the origami robot.

Referring to the movement of a measuring worm, we realized forward movement by changing the friction surface in the form shown in Figure 15.

In Figure 15, we show the layout of the BMX on the robot and the corresponding image between the activated and nonactivated BMX on the robot and the walking process. As shown in Figure 15, the BMX was placed on the robot to bend the robot body. In the left figure in Figure 15, the activated BMX and nonactivated BMX are represented by red and black lines, respectively. By controlling the BMX, we can control the friction force of the hind foot and the front foot.

To make the robot walk, we use the friction change of the ground surface during the walking motion of the robot. To make the origami robot walk, we set the BMX to the center place to bend the robot body as shown in Figure 15. We set steel wires to the robot legs to fix the angle of the leg. The steel wires are colored blue in Figure 15. When we control the robot, the BMX is controlled as shown in Figure 15 to realize walking. By controlling the BMX, we can control the friction force of the hind foot and the front foot. To differentiate the friction between the hind and front legs, we attached two rubber bands to the robot’s hind legs and one to the front legs. The red circles in Figure 15 show the layout of the rubber bands. When both feet of the robot are on the ground, the hind foot has more friction with the ground than the front foot. When the BMX is activated, the robot body is bent. In this case, the contact area of the hind foot decreases, and the hind foot has less friction with the ground than the front foot. As a result, the hind foot of the robot moves forward. On the other hand, when the BMX is deactivated, the robot’s body stretches back. In this case, the contact area of the hind foot increases and the hind foot has more friction with the ground than the front foot. As a result, the front foot of the robot moves forward. The robot moves forward by repeating the above actions.

As shown in Figure 15, two surfaces with different frictional forces were attached to both ends of the device. When the body is folded, the friction surface of the rear foot is farther from the ground. Therefore, the friction force of the front foot side becomes larger, and the rear foot can be pulled while the front foot is fixed.

On the other hand, when the robot stretches its body, the frictional force of the hind legs becomes larger, so that the hind legs are fixed and the front legs come forward during the stretching process. This repetition causes the robot to move as shown in Figure 15.

### 3.5. Robot Overview

**Figure 16:**
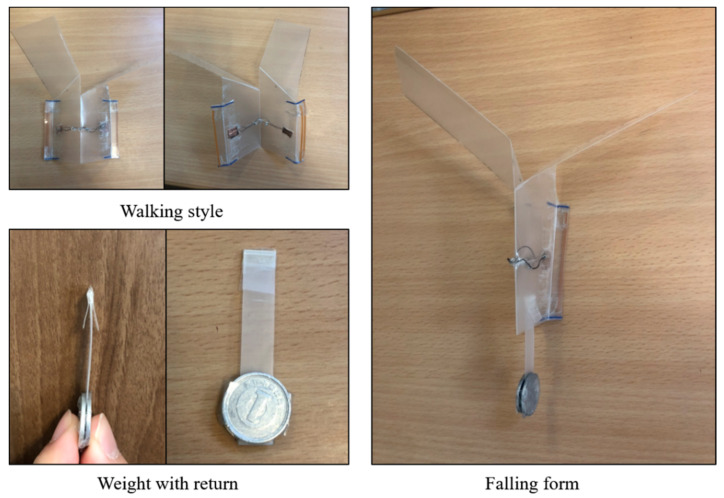
Overview of the developed sheet type robot.

Referring to the movement of a measuring worm, we realized forward movement by our origami robot as shown in Figure 16. The robot consists of a PP board with a single fold and a slit. The body part is 6.5 cm long and 5 cm wide, and the wings are 9.5 cm long and 4 cm wide.

### 3.6. Motion Experiment

We measured the dropping speed and moving speed of the origami robot by video to evaluate the validity and practicality of the structure. In order to measure the walking speed of the robot, a ruler was placed beside the robot. The time it took for the robot to walk a certain distance was analyzed by image analysis for video.

**Figure 17:**
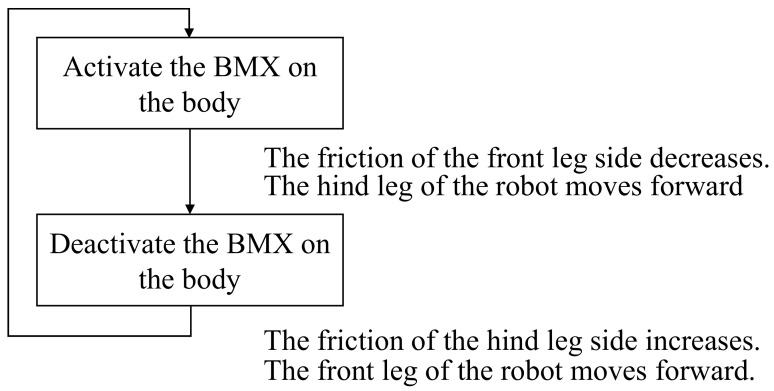
Walking diagram of how the sheet type robot walks.

**Figure 18:**
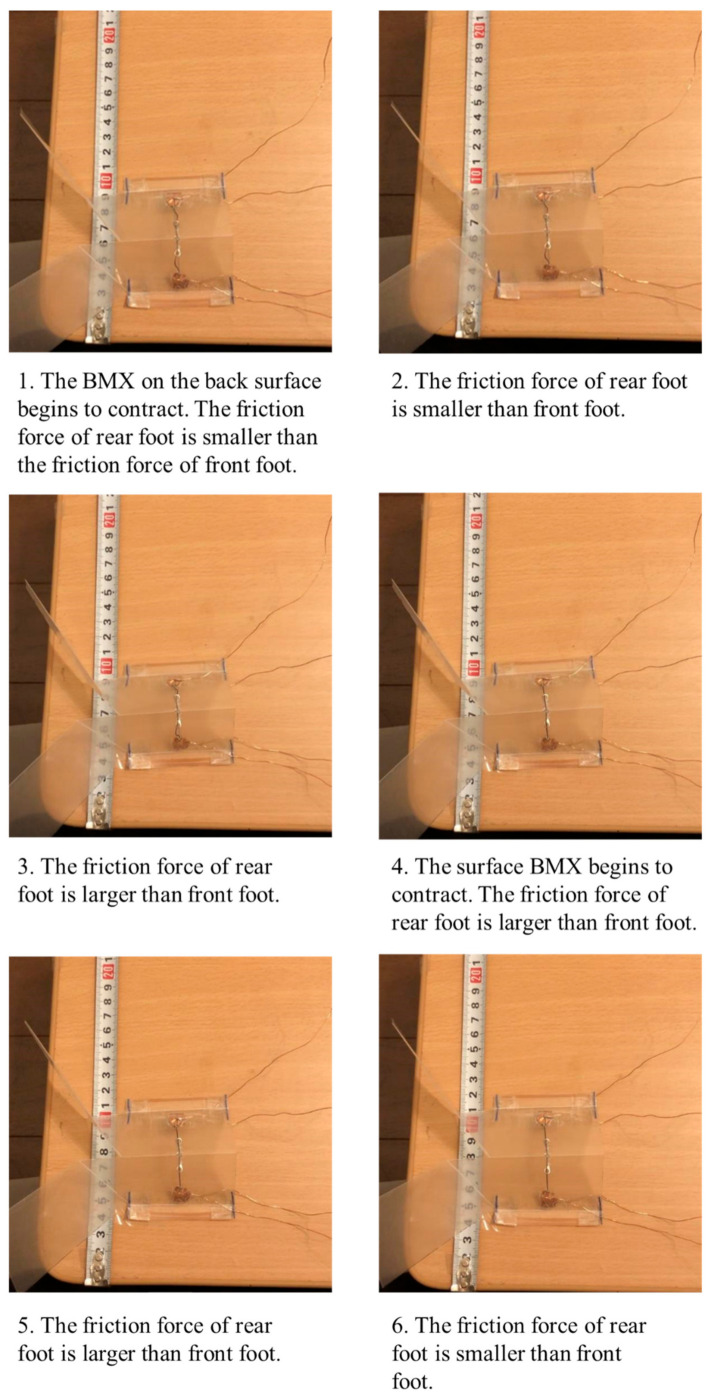
Walking experiment of the sheet type robot.

**Figure 19:**
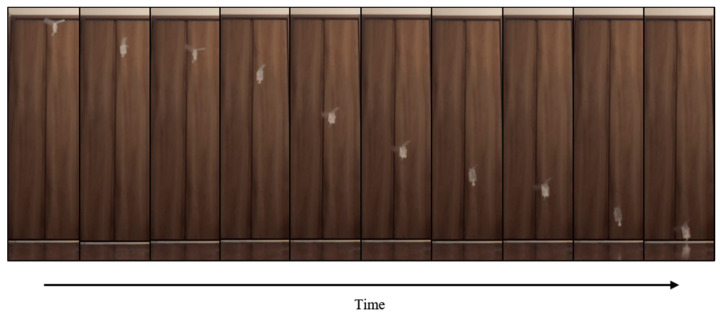
Drop experiment of the sheet type robot.

**Figure 20:**
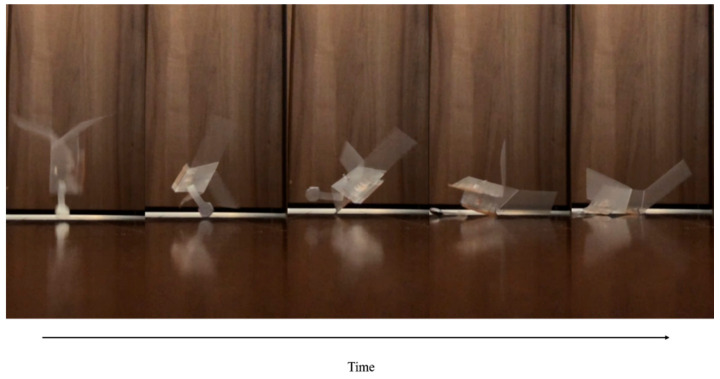
Deformation due to the weight detachment.

**Figure 21:**
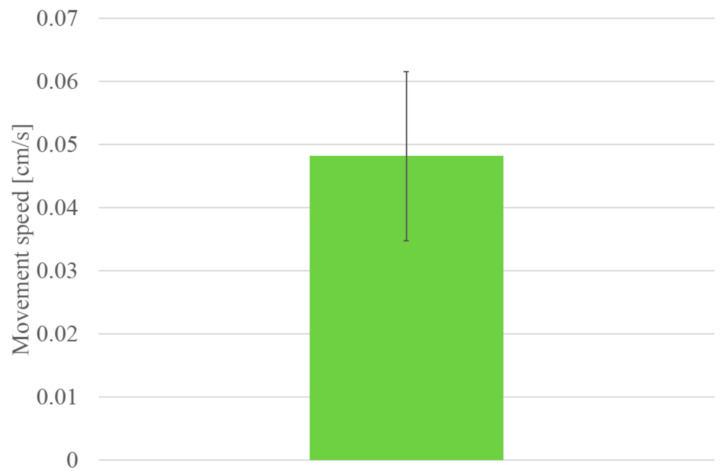
Experimental results of movement speed.

**Figure 22:**
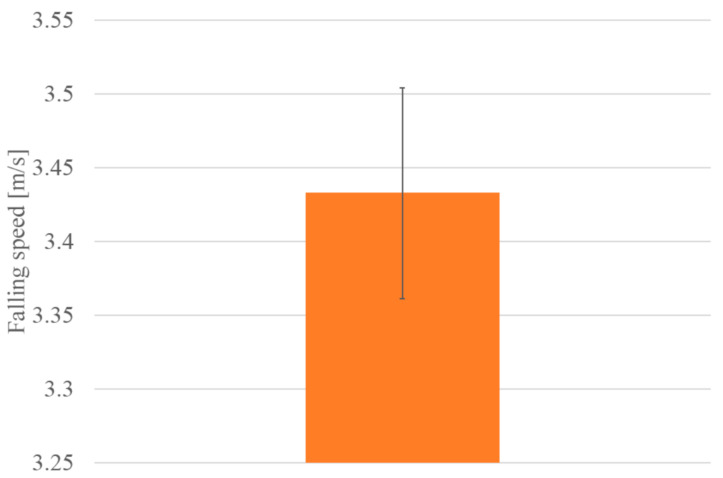
Experimental results of falling speed.

Figure 17 shows the walking diagram to show how the robot walks. As shown in Figure 17, repeated expansion and contraction of the BMX on the robot body changes the friction between the hind legs and front legs of the robot, resulting in forward movement. Figure 18 shows the walking experiment of the sheet type robot. Figure 19 shows the drop experiment of the sheet type robot. Figure 20 shows the deformation due to weight detachment. Figure 21 shows the experimental results regarding the movement speed on the ground. Figure 22 shows the experimental results regarding the falling speed from the sky. Each experiment was conducted five times, and the ± values represent the standard deviation. The moving speed was 0.0481 ± 0.0134 cm/s and the falling speed was 3.43 ± 0.0714 m/s.

## 4. Discussion

Through the motion experiments, we confirmed that the sheet type transformable robot can transform from a rotating fall to a walking form after landing, and that it can also walk on the ground. Based on the results of the movement experiments, we discuss falling, transformation and movement, respectively.

As for falling, we succeeded in reducing the speed of the model by rotating it, as in the case of the dipterocarp model. However, it is necessary to confirm whether the model works correctly when it is agitated by wind pressure from aircraft propellers or strong winds blowing in the sky.

By using a weight with a return, we were able to transform the robot from a falling form to a walking form without using electricity or actuators. At the same time, we were able to improve the walking performance by separating the weight that interferes with the movement. To check the transition between the flight mode and walking mode, we conducted five experiments on whether the robot could land in a walkable state after being dropped from the sky. Currently, the friction surface used in the walking mode is implemented only on one side. Therefore, if the device lands upside down, walking becomes impossible. We think that this problem will be solved by setting friction surfaces on both sides and allowing the BMX to bend in the opposite direction. We would like to improve the robot in the future to solve the problems.

The movement of the robot was realized with only one joint by devising the friction surface and the magnitude of the friction force, whereas it was realized with two joints in the past research [20]. In addition, the use of lightweight PP made it easy to bend the robot by BMX. However, turning and lateral movement could not be realized. As it is difficult to achieve with this structure, we need to consider a different folding method. Another problem is that the robot is not able to go over steps. In addition, the timing at which the friction surface touched the ground varied, and the moving speed was not stable. Although the experiment was conducted on a smooth table, the robot may not work properly depending on the ground condition because it walks by using frictional force.

## 5. Conclusions

In this study, we aimed to realize a transformable robot that could move after landing while reducing its falling speed by rotation after being thrown from the air. To achieve this goal, we developed a sheet type transformable robot that can transform from a falling form to a walking form.

In order to expand the functions of conventional disaster response robots to adapt to the environment, new parts must be added, which increase the size, weight, and cost of the device and complicate the control mechanism. Sheet type transformable robots have the potential to solve the problems of environmental adaptability and size enlargement because they can transform from a flat surface into various shapes and extend their functions. However, most of them have problems in moving to the disaster site for the purpose of disaster response because of their slow movement speed.

In order to solve these problems, we investigated the structure of a sheet type transformable robot with a simpler structure that can shorten the transportation time to the disaster site by aerial dispersal from the sky, while retaining its deformable extension. In this paper, we propose a new structure of origami robot that can shorten the transportation time to the disaster area by aerial dispersal.

We reported a developed prototype that can rotate, fall, deform, and move based on the mechanical model of a dipterocarp.

The developed origami robot has the following advantages.

It can be transformed from aerial spraying to walking form without using electricity or actuators due to its special shape. It is lightweight, thin, and small enough to be easily stored and transported. The contribution of this research with the above advantages is as follows.

We have proposed a robot that can be thrown from the air as a new transfer method to disaster areas that takes advantage of the softness and lightness of sheet type transformable robots.

In the future, we would like to consider the weight of the robot and the extensibility of its deformation, as well as the form of the fall, the method of movement, the materials and the actuators.

It is not preferable to drop the robots from the sky if a heavy object is loaded.

Therefore, in actual applications, we would like to use robots for searching for victims, rather than for carrying things. From this point of view, we would like to make it a size that can accommodate a small camera or microphone. In recent years, small cameras and microphones weighing 10 g or less have been put on the market. Considering these factors, we assume a robot that weighs several tens of grams in total.

Ultimately, we would like to realize a sheet type transformable robot that can search for people in distress, save people’s lives, and function as a useful tool in evacuation centers by itself after being thrown from the sky.

## Figures and Tables

**Table 1:** Falling velocity with respect to angle of attack and angular velocity.

Angle of Attack *α* (rad)	Falling Velocity (m/s)	Angular Velocity (rad/s)
2π/12	3.58	39.3
3π/12	3.47	56.7
4π/12	3.18	83.5
5π/12	2.98	69.2

## Data Availability

Not applicable.
